# Achieving Optimal Gestational Weight Gain, Birth Weight, and Perinatal Outcomes Among Pregnant Women at Risk of Hypertension: Protocol for a Pilot Randomized Controlled Trial

**DOI:** 10.2196/16676

**Published:** 2020-06-15

**Authors:** S M Tafsir Hasan, Syed Imran Ahmed, Md Alfazal Khan, Shafiqul Alam Sarker, Tahmeed Ahmed

**Affiliations:** 1 Nutrition and Clinical Services Division icddr,b Dhaka Bangladesh

**Keywords:** hypertensive disorder, hypertension, pregnancy, preeclampsia, gestational weight gain, continuous blood pressure monitor, wearable device, Health Gauge, birth weight, perinatal outcome

## Abstract

**Background:**

Hypertensive disorders, including preeclampsia, complicate 10% of all pregnancies, causing maternal and fetal morbidity and mortality. In Bangladesh, 24% of all maternal deaths are directly attributed to hypertensive disorders. Conventional antenatal care practices often delay or miss detecting hypertensive disorders in pregnancy, which may allow some women to become vulnerable to the adverse consequences of the hypertensive disorders. Regular self-monitoring of blood pressure and weight gain may improve maternal and fetal outcomes among pregnant women at risk of developing hypertensive disorders during pregnancy through early diagnosis, prompt referral, and timely clinical management; however, to undertake a randomized controlled trial of an intervention to reduce adverse consequences of hypertensive disorders in pregnancy, its feasibility must first be determined.

**Objective:**

The objectives of this study are to evaluate the accuracy of a wearable blood pressure monitoring device (Health Gauge) in order to test the design and methods of a future definitive randomized controlled trial, and to examine the feasibility, acceptability, and fidelity of an intervention focusing on regular monitoring of weight gain and self-monitoring of blood pressure for pregnant women at risk of developing hypertensive disorders and their associated complications.

**Methods:**

The study is located in Matlab, Bangladesh will be conducted in two phases. First, a wearable blood pressure device (Health Gauge) will be validated in accordance with the European Society of Hypertension International Protocol (revision 2010). Second, a prospective, two-arm, parallel, and nonblinded randomized controlled external pilot trial will be conducted. In the pilot trial, 70 eligible participants will be individually randomized to the intervention arm, in which pregnant women will self-monitor their blood pressure daily using a wearable device (Health Gauge) and be evaluated monthly by trained health workers for weight gain from 20 weeks of gestation until delivery, or the control arm, in which pregnant women will be assessed for weight gain every two months from 20 weeks of gestation until delivery (1:1 intervention to control allocation ratio using a permuted block randomization method with concealment). All women will receive standard antenatal care.

**Results:**

A validation study of the wearable blood pressure device has successfully been conducted among the general adult population in Matlab, Bangladesh. As of September 2019, the pilot trial has completed enrollment of women who are pregnant (N=70; intervention: n=35; control: n=35) and follow-up of the participants is ongoing. Data analysis is expected to be completed by June 2020, and results are expected to be submitted for publication in August 2020.

**Conclusions:**

The findings of this study will help us to design a comprehensive, full-scale randomized controlled trial to test the efficacy of regular self-monitoring of blood pressure and weight gain during pregnancy, a simple and inexpensive intervention to help to achieve optimal maternal and fetal outcomes in pregnant women at risk of developing hypertensive disorders and their associated complications during pregnancy.

**Trial Registration:**

ClinicalTrials.gov NCT03858595; https://clinicaltrials.gov/ct2/show/NCT03858595

**International Registered Report Identifier (IRRID):**

DERR1-10.2196/16676

## Introduction

### Background

Hypertensive disorders complicate 10% of all pregnancies worldwide [1]. They include chronic hypertension (pre-existing or when detected prior to 20 weeks of gestation), white-coat hypertension (an elevated blood pressure at clinic but normal blood pressure at home), masked hypertension (normal blood pressure at clinic but elevated blood pressure at home), gestational hypertension (elevated blood pressure detected after 20 weeks but no other systemic manifestations), and preeclampsia (elevated blood pressure detected after 20 weeks with proteinuria or biochemical or hematological abnormalities) [2]. Hypertensive disorders during pregnancy lead to severe morbidity and long-term cardiovascular disability in pregnant women and their offspring and are responsible for 14% of all maternal deaths [3,4]. Hypertensive disorders during pregnancy also lead to fetal growth restriction, preterm birth, and increased perinatal mortality [3]. Preeclampsia, a particularly devastating form of hypertensive disorder, is responsible for approximately 70,000 maternal deaths and more than 500,000 perinatal deaths globally each year [2].

In Bangladesh, 24% of maternal deaths are attributable to hypertensive causes [5]. Although the maternal mortality ratio in Bangladesh declined by 40% between 2001 and 2010 [6], the maternal mortality ratio stalled thereafter. The ratio in 2016 of 196 maternal deaths per 100,000 live births was identical to the estimate in 2010. Furthermore, the cause-specific mortality ratio due to hypertensive disorders of pregnancy increased from 39 per 100,000 live births in 2010 to 46 per 100,000 live births in 2016 [5]. Additionally, the prevalence of fetal growth restriction in Bangladesh is manifested in 30.5% of births being small for gestational age which is among the highest in the world. The rate of preterm birth which is 14.1% [7] is also high. Reducing maternal mortality, small for gestational age births, and malnutrition in children under five years age in Bangladesh and other low- and middle-income countries are now the major concerns of many governments and international agencies [8]; however, the most tractable pathways for effective interventions to promote healthy pregnancy, gestational weight gain, and fetal growth remain uncertain.

**Figure 1:**
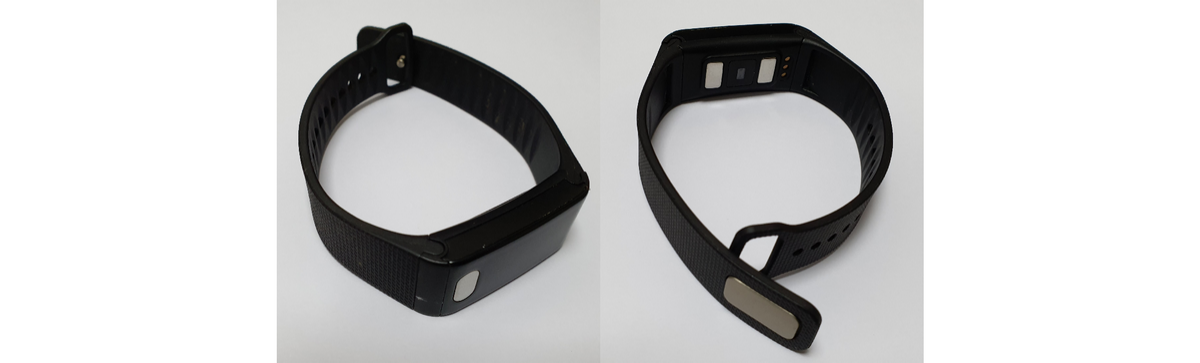
Health Gauge blood pressure monitoring device.

In conventional practice, pregnant women are evaluated for hypertensive disorders or associated complications during routine antenatal care visits. Standard antenatal care practices often delay or miss detecting hypertensive disorders during pregnancy, which may allow the women to become vulnerable to the adverse consequences of hypertensive disorders. Although, over the last two decades in Bangladesh, there has been an increase in the occurrence of at least one antenatal care (78%) visit during pregnancy, only 31% of women attend the recommended 4 antenatal care visits [6]. Furthermore, the quality of health care, which is fundamental to translating use of antenatal care services into improved maternal and fetal health outcomes, is generally poor in Bangladesh [5]. Weight and blood pressure measurements are two essential components of antenatal care services and should be ensured for quality care. Monitoring weight gain is important because both inadequate and excessive weight gain during pregnancy have been associated with an increased risk of developing hypertensive disorders [9,10]. To achieve an optimal pregnancy outcome, the United States Institute of Medicine provided recommendations for gestational weight gain stratified according to prepregnancy body mass index (BMI). The 2009 US Institute of Medicine guidelines on total gestational weight gain suggest that women who are underweight (BMI less than 18.5 kg/m^2^), normal weight (BMI from 18.5 to 24.9 kg/m^2^), overweight (BMI from 25.0 to 29.9 kg/m^2^) or obese (BMI greater than or equal to 30.0 kg/m^2^) should gain from 12.5 to 18 kg, 11.5 to 16 kg, 7 to 11.5 kg, and 5 to 9 kg, respectively. The recommendations for rates of weight gain during the 2nd and 3rd trimester are 0.51 (range 0.44-0.58) kg/week, 0.42 (range 0.35-0.50) kg/week, 0.28 (range 0.23-0.33) kg/week and 0.22 (range 0.17-0.27) kg/week, respectively, for the same BMI stratification [11]. As blood pressure has a property of circadian and other temporal variations, the current blood pressure measurement practices, which are also subject to interclinician and terminal digit bias, result in misdiagnosis [12]. A better method of hypertension diagnosis in women makes use of multiple measurements taken over continuous monitoring of blood pressure. While 24-hour ambulatory blood pressure monitoring could be an option, it can cause discomfort, particularly during pregnancy and is limited to only 24 to 48 hours of continuous monitoring [13]. In contrast, physiological parameter–derived blood pressure measurement is novel, noninvasive, and convenient to regularly monitor blood pressure. Blood pressure is estimated through linear modeling of extracted physiological parameters and machine learning algorithms that use features such as electrocardiogram (ECG) and photoplethysmography (PPG) derived pulse width and pulse transit time. Health Gauge is an affordable, wrist-worn wearable device that uses pulse width and pulse transit time to provide blood pressure measurements and is convenient for obtaining a picture of blood pressure and heart rate variability over time (Figure 1). Other methods, such as uterine artery Doppler ultrasonography and maternal blood concentrations of angiogenic factors and metabolomics, are also available to predict and monitor hypertension in pregnancy [14]; however, these tools are more expensive and less convenient for use in resource-poor settings including Bangladesh.

As the optimal management of hypertensive disorders of pregnancy remains unclear, the International Society for the Study of Hypertension in Pregnancy recommends that “*every hypertensive pregnant woman be offered an opportunity to participate in research, clinical trials, and follow-up studies*” [2]. We believe a simple intervention such as regular self-monitoring of blood pressure and weight gain has the potential to improve gestational weight gain and perinatal outcomes in pregnant women at risk of developing hypertensive disorders and complications through early diagnosis, prompt referral, and timely management. To undertake a randomized controlled trial of an intervention to reduce adverse consequences of hypertensive disorders during pregnancy raises important practical concerns about the implementation of the study. This exploratory study will address the question of whether a randomized controlled trial is an appropriate design and if it is feasible.

### Objectives

The aims of the pilot trial are to test the design and methods of a future definitive randomized controlled trial and to examine the feasibility, acceptability, and fidelity of an intervention focusing on regular monitoring of weight gain (once a month) during pregnancy using a digital weighing scale and regular self-monitoring of blood pressure (twice daily) using a wrist-worn blood pressure measuring device (ie, Health Gauge) in a sample of pregnant women who are at risk of developing hypertensive disorders and its associated complications. We will also evaluate the accuracy of Health Gauge in measuring blood pressure through validation studies. Acceptability and usability of Health Gauge for continuous self-monitoring of blood pressure in pregnancy will be assessed in the pilot trial.

## Methods

### Overview

The study will be conducted in two phases in Matlab, Bangladesh. Matlab is a low-lying riverine area which is situated 55 km southeast of the capital of Bangladesh. Since 1966, the icddr,b has been running an internationally recognized Health and Demographic Surveillance System involving a population of 230,000 in Matlab, Bangladesh [15]. Registration of vital events such as births, deaths, marriages, and migration are updated by community health research workers every two months. Information on reproductive health outcomes, contraceptive use, breastfeeding, and immunization is also collected.

### Phase 1: Validation of Health Gauge

The Health Gauge device and an artificial intelligence and machine learning–based companion smartphone app will be provided by Salu Design Group Inc. Health Gauge is a compact, wrist-based, cuff-less blood pressure monitoring device that uses a combination of 2-contact ECG and PPG as well as a combination of pulse wave analysis algorithms, machine learning, and neural network computing techniques to calculate blood pressure instantly. The blood pressure data can be synchronized with and accessed from a smartphone through the secure-access app.

In internal quality control preliminary tests, Health Gauge provided precise and accurate blood pressure measurements in adults (personal communication, Randy Duguay, CEO, Salu Design Group Inc); however, further evaluation is required in our setting to determine whether the device functions effectively and records blood pressure measurements accurately. The validation study aims to assess the accuracy of the device in measuring blood pressure, according to the European Society of Hypertension International Protocol (ESH-IP revision 2010) [16].

A total of 33 participants who fulfill the age, sex, baseline blood pressure, and other requirements will be included in the validation study. For age criteria at least 10 men and 10 women who are 25 years or older, and for baseline blood pressure, 10 to 12 participants with systolic blood pressure in each of three ranges (90 mmHg to 129 mmHg, 130 mmHg to 160 mmHg, and 161 mmHg to 180 mmHg) and 10 to 12 participants with diastolic blood pressure in each of three ranges (40 mmHg to 79 mmHg, 80 mmHg to 100 mmHg, and 101 mmHg to 130 mmHg) will be included. Validation in specific groups such as adolescents or pregnant women may also be carried out with necessary modification of these requirements, and all such changes or additions will be clearly described during reporting. All the participants will be recruited from outpatients of Matlab hospital (icddr,b) or as volunteers residing in Matlab near the hospital.

The validation team will consist of three trained medical doctors (two observers and one supervisor). The gold standard reference blood pressure measurement will use two standard mercury sphygmomanometers and a good quality teaching stethoscope. Simultaneous auscultations will be performed by two observers using the teaching stethoscope. These two observers will be blinded to each other’s readings. The supervisor will verify the blood pressure readings of the other two observers to ensure that the difference between the two observations is less than or equal to 4 mmHg for either systolic or diastolic pressure values. If the difference is greater than 4 mmHg for either, the measurement will be repeated. The supervisor will also measure blood pressure using Health Gauge. The blood pressure measurements will be alternated between the mercury sphygmomanometer and Health Gauge device. In total, nine consecutive blood pressure measurements will be performed in each participant using the mercury sphygmomanometer (5 times) and Health Gauge (4 times).

Data will be analyzed and reported according to the ESH-IP revision 2010 requirements to conclude if Health Gauge passes the validation protocol; the differences between the measurements obtained from Health Gauge and the mercury sphygmomanometer will be classified according to whether the values are within 5 mmHg, 10 mmHg, or 15 mmHg. The differences will be classified separately for systolic and diastolic blood pressure. Details of the methods, procedures, and analysis have been described elsewhere [16,17]. Phase 2 will proceed when Health Gauge has been validated.

### Phase 2: Pilot Randomized Controlled Trial

#### Study Design

This study is designed as a prospective, two-arm, parallel, and nonblinded randomized controlled external pilot trial. Eligible participants will be individually randomized in a 1:1 allocation ratio to the intervention arm, in which pregnant women will self-monitor their blood pressure daily using a wearable device (Health Gauge) and be evaluated for weight gain monthly from 20 weeks of gestation until delivery, or the control arm, in which pregnant women will be assessed for weight gain every two months from 20 weeks of gestation until delivery. All women will receive standard antenatal care.

#### Study Population Eligibility Criteria

Inclusion criteria are pregnant women in Matlab, Bangladesh with a high-risk pregnancy who are between 12 and 16 weeks of gestation and are between 15 and 50 years of age. Exclusion criteria are women who have a congenital malformation or anomaly, a chromosomal abnormality (such as Down syndrome), chronic debilitating illness, diagnosed psychosis, no electricity at home, and never used a smartphone. In this study, a high-risk pregnancy is defined to identify women at risk of developing hypertensive disorders of pregnancy or their complications [3], as a pregnant woman who meets any one or more of the following criteria: had preeclampsia or gestational hypertension in a previous pregnancy, has chronic hypertension, has chronic kidney disease, had pregestational diabetes, has systemic lupus erythematosus or antiphospholipid syndrome, pregnancy is her first pregnancy (nulliparity), is aged 40 years or older, interpregnancy interval is less than 2 years or greater than 10 years, has a BMI of 35 kg/m^2^ or more, has polycystic ovary syndrome, has a family history of preeclampsia, pregnancy is a multiple pregnancy, has pre-existing thrombophilia, used of selective serotonin reuptake inhibitors beyond the first trimester, has donated a kidney, underwent in vitro fertilization, and has a family history of coronary heart disease.

#### Sample Size

Since this is an exploratory study, conventional sample size calculation may not be applicable; however, we will aim for 35 participants in each arm based on the recommendation by Whitehead et al [18] on the required sample size for pilot trials and by considering a conservative (small) effect size for the definitive trial designed with 90% power and two-sided 5% significance. Our sample size calculation takes into account a 20% attrition.

#### Randomization and Allocation Concealment

Participants will be assigned to the intervention or the control arm using a permuted block randomization method with concealment to ensure that the allocation is not made before the participant has given their consent and joined the study. A random allocation sequence will be generated using a computerized random allocation system (RALLOC module in Stata statistical software; version 14.1; StataCorp LLC) for permuted block randomization to ensure comparable allocation numbers at a certain equally spaced points in the sequence of patient assignment and randomization for parallel study design will be used. Reasonably large blocks with variable block size will be constructed to reduce predictability. The random allocation sequence will be prepared in advance by an independent researcher from icddr,b who has no involvement with the trial.

The randomization list will be transferred to health workers via the health research supervisor into sequentially numbered nontransparent sealed envelopes each containing the name of the group (intervention or control) on a card inside the envelope to ensure that the study personnel do not know the order of this list and are unable to predict the next assignment/allocation. Only one block will be supplied at a time from the independent researcher, and the next block will be provided just after completion of the previous block. The envelopes will be kept in a locker in a secured place, and the key of the locker will be available with the health research supervisor. The health research supervisor will not use the key to open the lock until the last envelope given to health worker has been used. The independent researcher will keep a duplicate set of sealed envelopes.

#### Blinding

Neither the participants nor the investigators and assessors of outcomes can be blinded to allocation because of the nature of service delivery that will be provided, and thus, we are obliged to make the randomized controlled trial nonblinded; however, it should be mentioned that the outcomes of the trial are objective in nature, therefore, the risk of bias is minimal. Furthermore, the randomization, as well as statistical analysis, will be carried out by someone unconnected to the enrollment process.

#### Intervention

Before 20 weeks of gestation, each woman in the intervention arm will be provided with a Health Gauge device and with a smartphone, if she does not already have one. The app for Health Gauge will be installed on each participant’s smartphone. The women will be asked to use the Health Gauge device and app to measure their blood pressure at least two times a day (morning and evening), starting from 20 weeks of gestation. The blood pressure measurement records will be automatically stored in the smartphone app. Participants will be trained to charge and operate the Health Gauge device. Most women in rural Matlab do not have internet access. Hence, trained health workers will visit the women weekly and synchronize the smartphones with tablet computers to collect the stored data and to upload the data to an online server. The health workers will also measure the weight of the participant using a digital weighing scale every month. If any woman is found to be hypertensive (blood pressure is greater than or equal to 140/90 mmHg on two consecutive measurements), is presenting signs and symptoms associated with hypertensive disorders or associated complications (described later), or is gaining weight outside of the applicable US Institute of Medicine recommended range [11], she will be advised to visit Matlab hospital (icddr,b) or a government health facility immediately for further evaluation and management. If the condition demands, our health worker will ensure that an appropriate referral is made to a tertiary health care facility. This intervention will be in addition to the conventional programs offered by icddr,b as well as government health facilities.

In conventional care, women without any major risk factors, danger signs, or health conditions should visit a health facility four times during the antenatal period (at 8 to 12 weeks, 24 to 26 weeks, 32 weeks, and 36 to 38 weeks, with an additional visit at 41 weeks if they have not yet given birth). Pregnant women are separated into two groups by their care requirements—those eligible to receive routine care and those who require special care based on their health conditions or risk factors. Women with risk factors or special conditions are referred to a specialized clinic or hospital for further evaluation, and care is continued according to a specialist's advice. Women who are initially referred to a specialist may be subsequently considered eligible for routine care and women who are initially enrolled in routine care may later need to be referred for specialized care [19].

#### Control

All women randomized to the control arm will receive standard antenatal care. In addition, health workers will visit the women every two months starting from 20 weeks of gestation to measure their weight using a digital weighing scale. If any woman is gaining weight outside of the applicable US Institute of Medicine recommended range, she will be advised to visit Matlab hospital (icddr,b) or a government health facility immediately for further evaluation and management. If the condition demands, our health workers will ensure that an appropriate referral is made to a tertiary health care facility.

Health workers will counsel all participants, both in the intervention and in the control group, to make at least four antenatal visits. All women will be provided with general education to maintain a balanced diet and to remain physically active during pregnancy.

#### Participant Enrollment and Follow-up

In Matlab, pregnancies are usually diagnosed by 12 weeks of gestation and recorded by Health and Demographic Surveillance System field staff. We will obtain this information from Health and Demographic Surveillance System and conduct baseline interviews to identify eligible participants for this study. Health worker will continue to conduct baseline interviews and enroll participants whenever newly pregnant women are identified, and to obtain information from the Health and Demographic Surveillance System until the desired sample size is met. Each potential participant will be provided with a consent form in the local language (Bengali). Health worker will also explain the study in detail and answer any questions. If a woman agrees to participate in the study, her signature or left thumb impression and that of a witness will be taken. A health worker will also sign the consent form in front of the woman and the witness on behalf of the principal investigator of the study. Then the health worker will conduct a short interview using a semistructured questionnaire and record the woman’s height and weight. If eligibility criteria are satisfied, the health worker will open the sealed envelope (in sequence and in front of the participant and the witness) that assigns the participant to the allocated group (intervention or control). If the woman does not meet the eligibility criteria, she will be informed that she cannot take part in the study.

Once enrolled, the women will be followed from 20 weeks of pregnancy until delivery. Health worker will visit women in the intervention arm weekly and those in the control arm every two months. Furthermore, health workers will maintain mobile communication with all women to keep track of the progress of the pregnancy. Health worker will collect outcome data from the households and health facilities by follow-up visit or by phone.

#### Data Collection

##### Questionnaire Preparation

Questionnaires will be prepared in English, and then translated into Bengali with back-translation. The Bengali questionnaires will be used in the field. Pretesting will be done before finalizing the questionnaire. Field staff will be given training on administering the questionnaires.

##### Baseline Questionnaire

A brief questionnaire will be designed to collect baseline information on the participants. Questions on age, education level, occupation, and work status of the pregnant woman, her husband, and members in her household will be included. Information on household income, marital status, parity, whether pregnancy was intended, living conditions, tobacco use, health and nutrition, knowledge of nutrition and dietary practices, health-seeking behavior, and media exposure will also be collected.

##### Follow-up Questionnaire

Health worker will administer a follow-up questionnaire weekly to the women in the intervention group to collect information on signs and symptoms associated with hypertensive disorders and complications, including sense of awareness, headache, blurring of vision, abdominal pain, nausea and vomiting, edema, and convulsions over the previous week.

##### Acceptability

The acceptability of using Health Gauge for continuous self-monitoring of blood pressure will be evaluated by women in the intervention group using a 5-point Likert scale. In addition, interviews will be conducted to assess the women’s perception two times: during the third trimester and after delivery.

##### Perinatal Outcomes

Information including predelivery weight, perinatal outcomes, and infant length, weight, and head circumference at birth will be obtained from hospital records, discharge certificates given after institutional delivery or from the woman herself using a simple checklist.

##### Health and Demographic Surveillance System Records

Age and asset score (wealth quintile) of the study participants will be retrieved from the Health and Demographic Surveillance System database.

##### Anthropometry

Health worker will measure the height and weight of each pregnant women following standard anthropometric procedures. Height will be measured using locally made standardized stadiometer. Weight will be measured using a digital weighing scale.

#### Training of the Health Workers Delivering the Intervention

Four skilled and experienced health workers who each have at least 12 years of formal education will undergo a fifteen-day intensive training program, which will include lectures, mock interviews, role play, and field practice at the community level. A training manual will be developed to guide the health workers in the field.

#### Data Quality

To ensure the accuracy of the data, several quality control measures will be undertaken at different stages of the data collection procedure: (1) Since the participants are women, we will recruit experienced female health workers for data collection to minimize observer bias. (2) We will establish a multilayered monitoring system to maintain and standardize data quality. A health research supervisor will check data consistency daily by thoroughly checking the filled-out questionnaires at the end of the day. (3) We will perform spot-checking, re-interview, back-checking, and provide necessary feedback. (4) Regular meetings with the research team and refresher training sessions (if required) will be arranged for the health workers.

#### Outcome Measures

##### Primary

The feasibility outcomes (primary outcomes) will be recruitment rate, retention rate, adherence to the protocol, and acceptability. Recruitment rate will be defined as the mean number of participants recruited per month. Both eligibility and consent rate will be recorded. Recruitment rate is an important outcome because unforeseen enrolment challenges may arise and are crucial to identify in a pilot study. Retention rate will be defined as the proportion of participants who will participate in the full follow-up period of the study (from 20 weeks of gestation until delivery). Adherence will be defined as the proportion of participants following the intervention protocol. There may be several reasons why a participant does not follow the intervention protocol properly. Health workers will note the cause and the number of instances of nonadherence in a log sheet. Nonadherence will be classified into protocol deviations caused by circumstances beyond their control and those that are not (for example, the participant chose to remove Health Gauge). Participant will rate the acceptability of using Health Gauge for continuous self-monitoring of blood pressure two times during the follow-up period using a 5-point Likert scale. Acceptability will be evaluated once during the third trimester and again after delivery.

##### Secondary

Clinical outcomes will be rate of weight gain during pregnancy; infant weight, length, and head circumference at birth; hypertension status; adverse consequences of hypertensive disorders; blood pressure profile and heart rate; risk factors; and serious adverse events. Rate of weight gain during pregnancy will be measured in kilograms per week and be calculated by subtracting the baseline (enrollment) weight from the predelivery weight and dividing that value by the number of weeks between the two time points. Infant weight will be measured in grams, infant length will be measured in centimeters, and head circumference at birth will be measured in centimeters. The number of women with chronic hypertension and who develop hypertensive disorders will be recorded. Adverse consequences of hypertensive disorders of pregnancy will be defined as maternal or fetal complications or consequences that arise in a woman with chronic hypertension or in a previously normotensive woman who has developed some form of hypertensive disorder after at least 7 days of intervention. Maternal complications are abruptio placentae, disseminated coagulopathy or hemolysis, elevated liver enzymes and low platelets syndrome, pulmonary edema, acute kidney injury, eclampsia, liver failure or hemorrhage, stroke, or death. Fetal or neonatal complications are intrauterine death or stillbirth, preterm birth, small for gestational age, low birth weight, hypoxic brain injury, and complications associated with small for gestational age, low birth weight, or prematurity. Episodes, type, and timing of occurrence of adverse consequences will be reported. The blood pressure profile along with heart rate from 20 weeks of pregnancy until delivery will be recorded. The prevalence of specific risk factors along with the sociodemographic profile of the participants will be determined. We do not anticipate any serious adverse events in this trial; however, should any other adverse events arise they will be reported in detail.

#### Covariates

Covariates in this study are age (in years), height (in centimeters), weight (in kilograms), body mass index (in kg/m^2^), parity, duration of pregnancy, maternal smoking (including second-hand smoke) and use of chewing tobacco, and asset score (socioeconomic status).

#### Data Analysis

Data presentation, analysis, and reporting will be carried out according to CONSORT randomized pilot and feasibility trial guidelines [20]. The feasibility outcomes will be reported descriptively as well as narratively. Only descriptive statistics will be reported for the clinical outcomes. Mean and 95% confidence interval will be reported for continuous variables, median and interquartile range will be reported for ordinal variables, and raw count (number and percentage) will be reported for categorical variables. No conventional test of hypothesis will be performed since as a pilot trial, statistical power is lacking; however, the estimates of effect using clinical outcomes as they are likely to be measured in the definitive trial will be reported as estimates with 95% confidence interval. Analyses will be performed at the end of the study. No interim analyses or subgroup analyses are planned due to the short duration and small sample size of this pilot trial. Data will be analyzed using Stata software (version 14.1; StataCorp LLC).

#### Ethics Approval

The study was reviewed and approved by the Research Review Committee and the Ethical Review Committee of icddr,b (PR-18026). This study has been registered with ClinicalTrials.gov (NCT03858595).

## Results

Phase 1, the validation of Health Gauge, was successfully conducted among general adult participants of both sexes in Matlab, Bangladesh. Validation among special groups is underway. As of September 2019, the pilot trial (phase 2) has completed enrollment of women who are pregnant (N=70; intervention: n=35; control: n=35). Follow-up of the participants is ongoing and is expected to be completed by the end of October 2019. Data analysis is expected to be completed by June 2020, and results are expected to be submitted for publication in August 2020.

## Discussion

In this protocol, we present the design and procedures of a two-phase experimental study in which we validate an intervention tool (Health Gauge, a wrist-worn blood pressure measuring device) and evaluate the feasibility of an intervention (regular self-monitoring of blood pressure by Health Gauge and health worker–guided monitoring of weight gain during pregnancy using a digital weighing scale) through a pilot trial. To our knowledge, this is the first study of its kind in Bangladesh and worldwide. In this study, we focus on a well-defined at-risk population (pregnant women at risk of developing hypertensive disorders of pregnancy or their complications) with an urgent need for accessible, low-cost measures to mitigate the adverse consequences of hypertensive disorders of pregnancy. We conjecture that a simple intervention such as regular self-monitoring of both blood pressure and weight gain could be useful for improving maternal and fetal outcomes in this population through early diagnosis, prompt referral, and timely management. The proposed intervention will be tested for efficacy through a future randomized controlled trial, and this feasibility study will guide the definitive randomized controlled trial. This study provides a platform for both developing and testing a new technology for self-measurement of blood pressure among end-users. The findings will shed light on the acceptability of the device and appropriate measurement practices among the target population. The findings will also guide changes and improvements in the device, its companion app, and the monitoring protocol. That our research team consists of people from diverse background with expertise in maternal and child health and nutrition, clinical and public health research, trial design, and computer programming and machine learning algorithms will help us adopt a multidisciplinary and comprehensive approach to achieve a common goal—healthy pregnancy, healthy baby.

A major limitation of this study is that pregnant women without access to electricity at home or who are not familiar with smartphones must be excluded. Additionally, limited internet access to the internet among participants as a result of Matlab’s rural setting makes live monitoring of blood pressure data difficult. Access to the blood pressure data will only be available at weekly intervals when the health workers visit the participants and synchronize the data with the online server. Nevertheless, pregnant women will be trained to take their own blood pressure measurements using Health Gauge. They will be informed to contact the health workers by phone and visit the nearest health facility if they find that their blood pressure is greater than or equal to 140/90 mmHg on two consecutive measurements. Another limitation is the lack of nonsubjective qualitative approach to ascertain acceptability and usefulness of the intervention. Finally, the small sample size limits the statistical power to detect any effect of the intervention on clinical outcomes; however, the sample size should be sufficient to accomplish the objectives of this pilot trial.

After successful completion of this study, the data will be accumulated, analyzed, and reported. The companion app for Health Gauge will be evaluated and improved using data generated in the study and machine learning for more accurate and precise blood pressure measurements; the design of Health Gauge may also be modified or adapted, if necessary. In the definitive trial, the intervention may include additional educational materials and focused counseling sessions. The implementation protocol may be revised to allow women to self-monitor weight gain akin to the protocol used for blood pressure measurements. Based on the results of and experience gained from this pilot trial, we will design a full-scale randomized controlled trial to test the efficacy of the intervention.

## Abbreviations

ECG: electrocardiogram
ESH-IP: European Society of Hypertension International Protocol
icddr,b: International Centre for Diarrhoeal Disease Research, Bangladesh
PPG: photoplethysmography
